# Remembering Thomas J. Baker, MD

**DOI:** 10.1093/asjof/ojab006

**Published:** 2021-01-29

**Authors:** James M Stuzin

**Affiliations:** Department of Plastic Surgery, University of Leonard M. Miller School of Medicine, Miami, FL, USA

You only live once, but if you do it right, once is enough.

― Mae West

It is with great sadness that Thomas J. Baker, MD, (Figure 1) passed away on December 26, 2020, at the age of 95. A pioneer of aesthetic plastic surgery and a founding member of the American Society for Aesthetic Plastic Surgery (now known as The Aesthetic Society), Dr Baker enjoyed a long career of over 45 years, making significant contributions to the validity of aesthetic surgery as an important subspecialty of academic plastic surgery (Video).

**Figure 1.**
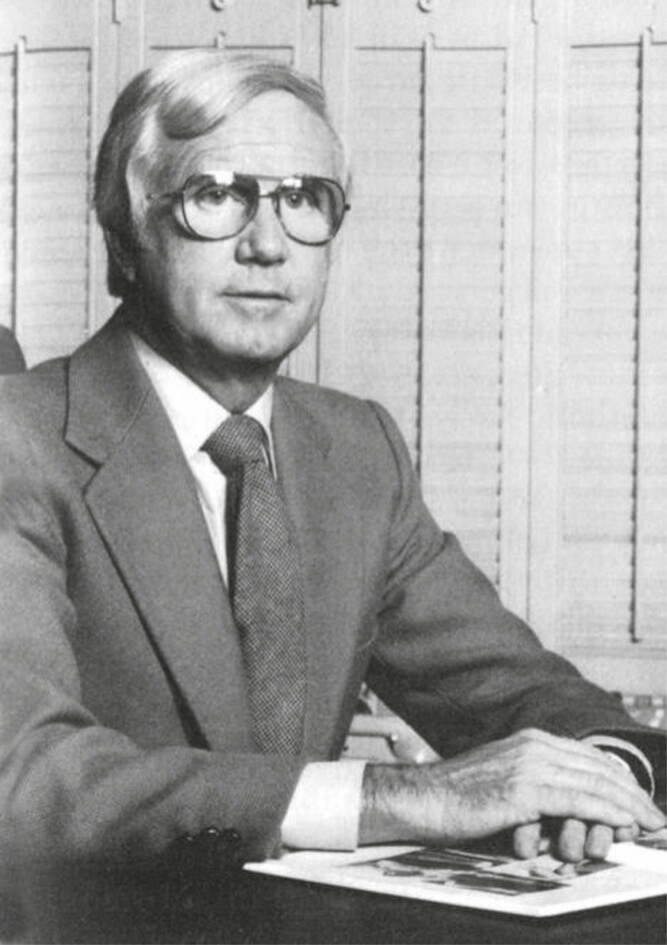
Thomas Baker, MD.

For me, writing this tribute is a difficult task, and losing someone you love is a great loss. Nonetheless, looking back also provides an opportunity to not only recognize accomplishment but also provide a means to celebrate a life well lived. And Tom Baker certainly lived a full and fulfilled life. 

Tom lived what some might term the “American Dream,” from humble beginnings to world-renown fame within plastic surgery. Tom was born on November 8, 1925, in the Midwest farm town of Clay, Kentucky, and was the only child of Emily and Thomas Baker. His father died when he was young and he was largely raised by his mother Emily (Figure 2), who lived to the age of 96. The family later moved to Boonville, Indiana, and, after graduating high school, he attended Indiana University for both undergraduate and medical school. After spending time in the Navy, Tom completed his general surgery training at the University of Miami and his plastic surgery training at the University of Texas Galveston, studying under Dr Truman Blocker. After a brief period spent teaching at the University of Missouri, Tom returned to Miami in 1959 and Dr Howard Gordon joined him in partnership a year later (Figure 3).

**Figure 2.**
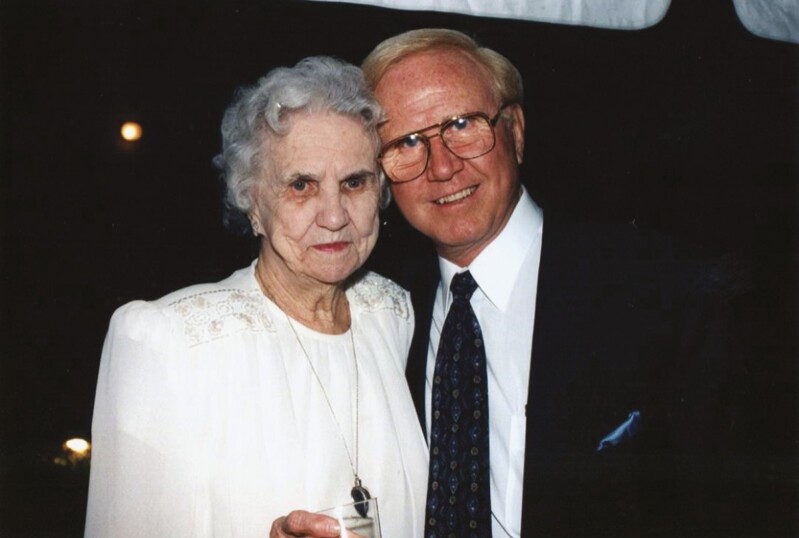
Thomas Baker with his mother, Emily.

**Figure 3.**
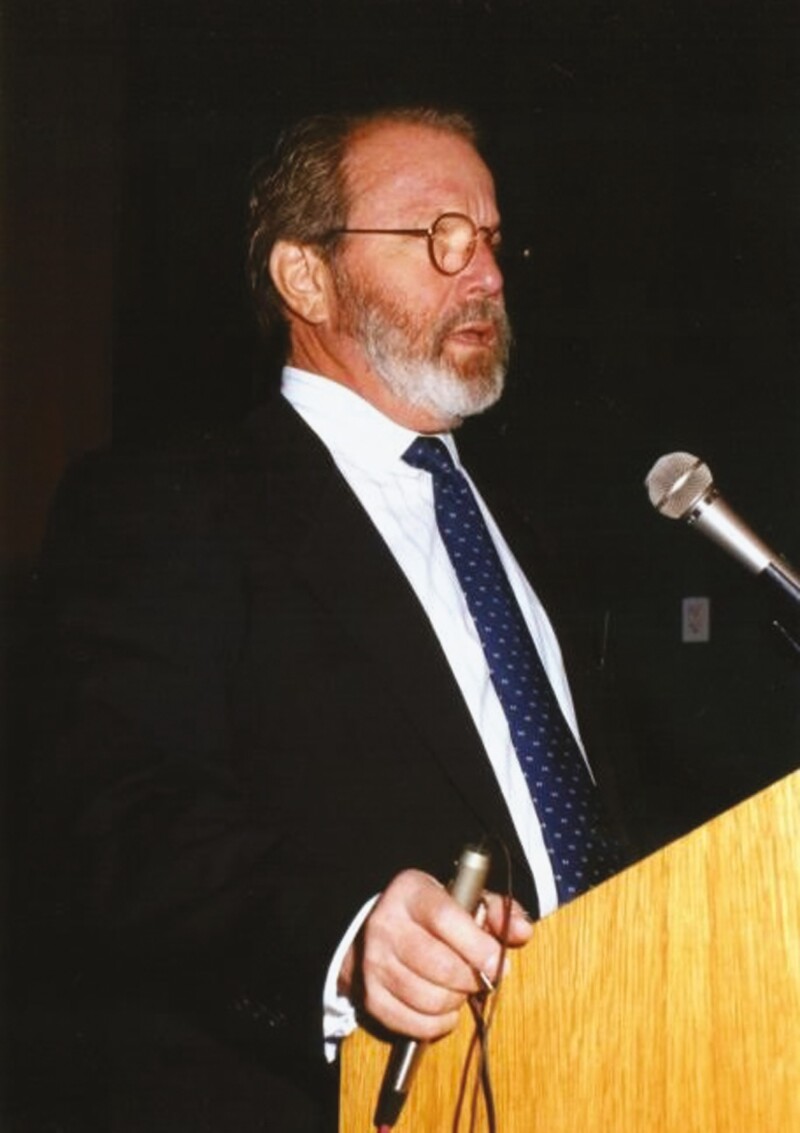
Howard Gordon, MD, lecturing at the Baker Gordon Symposium, in the early 1990s.

While Tom received no formal training in aesthetic surgery while at UT Galveston, both a personal interest and patient demand kindled a passion to learn how to perform aesthetic procedures safely and with expertise. He often told me that what began this journey was his amazement of the results of patients who had been treated in a lay clinic in North Miami (known as the House of Renaissance) undergoing a “secret formula” chemical peel that produced a total reversal of wrinkled photoaged skin. After visiting the clinic to try to learn the essence of the formula, as well as doing laboratory studies on rabbits regarding the safety of the procedure, he performed his first phenol peel to the forehead of an elderly patient, reasoning that if she scarred her forehead he knew how to reconstruct this aesthetic subunit following his burn reconstruction training at UT Galveston. Fortunately, she experienced uneventful healing. After repeating the procedure on numerous patients, Tom wrote the first scientific article on phenol peeling, published in Plastic and Reconstructive Surgery (PRS) in 1962, where he delineated what was later termed the Baker Gordon formula.^1^ This article was initially greeted with great skepticism by academia, but over time science won out over opinion, and other investigations regarding the science of resurfacing proliferated in the plastic surgery and dermatology literature. He also wrote seminal articles in *Aesthetic Surgery Journal *on approaches to midface and cheek lifting^2^ and an article on extended SMAS dissection for midface rejuvenation in *Clinical Plastic Surgery*.^3^

As his practice became more centered on aesthetic procedures, Tom became frustrated at the paucity of resources for aesthetic surgery education in the 1960s. During that era, there were a few recognized experts in the field, and these experts were largely reticent to “train” the competition. A university-based bias against aesthetic surgery led to few journal articles being published on aesthetic techniques, and there were limited textbooks on the subject. For this reason, Tom decided to invite recognized leaders in aesthetic surgery to Miami to educate their peers, founding the Baker Gordon Symposium in 1967. The first invited guests were Drs Salvadore Castanares and Thomas Rees, and the first Baker Gordon Symposium was held at Cedars Hospital, which later became the University of Miami Hospital. Bleachers were constructed and 20 participants observed live surgery directly in the operating room. Following the initial meeting, the demand to attend the Baker Gordon Symposium grew as plastic surgeons demonstrated their hunger for aesthetic surgery education. Under Tom’s guidance and friendship, the worlds’ experts on aesthetic surgery annually traveled to Miami, educating their peers to perform procedures safely and consistently (Figure 4).

**Figure 4.**
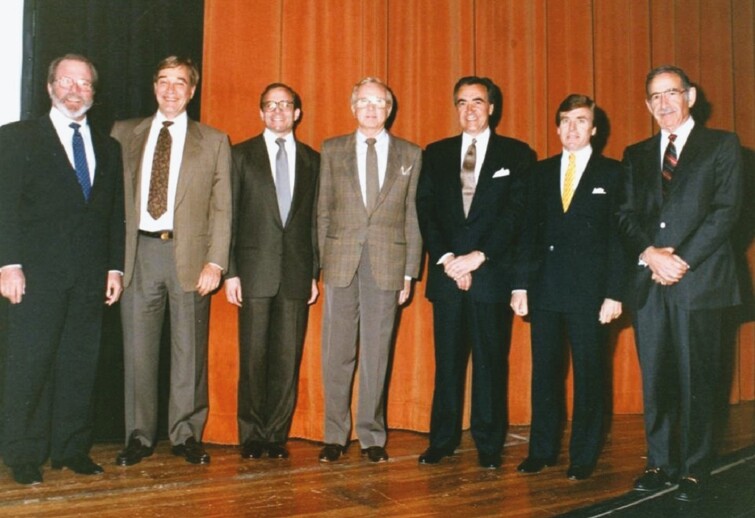
The faculty of the 25th Baker Gordon Symposium in 1991. (From left to right) Howard Gordon, MD; Bruce Connell, MD; James Stuzin, MD; Thomas Baker, MD; Jack Gunter, MD; Sherrell Aston, MD; and Jack Sheen, MD.

Tom became not only a recognized expert in aesthetic surgery but also through the Symposium, became renowned as an educator. Over the ensuing 55 years, The Baker Gordon Symposium has taught thousands of plastic surgeons to improve the quality of care they deliver to their patients, and the edited videos from this meeting remain the top videos views from the PRS streaming video platform to educated plastic surgeons globally on advances in aesthetic techniques. 

I entered practice with Tom in 1987, following completion of my residency and fellowship training (Figure 5). The first day I started with him, I assisted him in doing a face-lift. After completion of the subcutaneous dissection, Tom handed me the scalpel and said “Jim, do you think the superficial muscular aponeurotic system (SMAS) works to improve the results in face lifting. I haven’t really noticed a difference.” Having trained with surgeons, such as Drs Thomas Rees and Sherrell Aston, who routinely did some lateral SMAS undermining, he handed me the scalpel and told me to show him how to perform the dissection. After watching me do the SMAS dissection, he said, “you know, I really like that,” and he readily (and more dexterously) repeated the dissection on the contralateral cheek. Always embracing change and innovation, Tom then routinely incorporated SMAS dissection into his personal face-lift technique.

**Figure 5.**
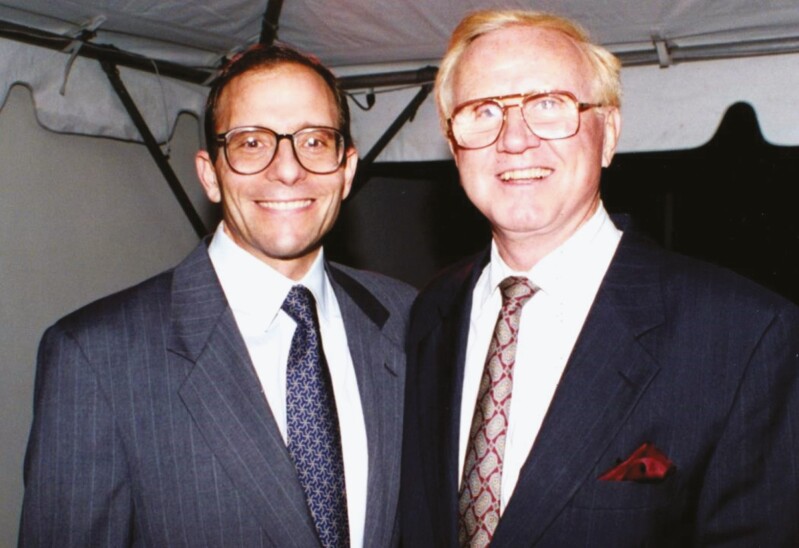
Drs Thomas Baker and James Stuzin at the 25th Baker Gordon Symposium, 1991.

Resulting from our desire to gain greater consistency in face lifting, Tom became interested in facial anatomy, which was incompletely delineated in the literature of that era. Tom was a pilot, and this allowed us to fly to the University of Florida, where we had access to fresh cadavers. Tom would closely watch as I did the dissections to better define the lamellar relationships that exist within the facial soft tissue architecture. These dissections confirmed for us that the concepts of Skoog and others allowed subSMAS dissection to be performed safely as long as basic anatomic concepts are understood. This then led us to incorporate more extensive subSMAS dissections into our face-lifting technique, which we termed the “extended SMAS” dissection, as it extended the standard lateral cheek dissection of that era medially toward the malar region.

Tom and I did our first “extended SMAS” dissection in 1989, on a patient who had facial weakness resulting from a Bell’s palsy. I did the dissection with Tom watching carefully over my shoulder and we were both amazed and relieved that the lamellar relationships we had seen in the cadaver translated safely into the operating room. In 1991, after having performed this dissection on a large number of patients, we demonstrated the “extended SMAS” technique at Baker Gordon. Over time, several variations of extended SMAS procedures have evolved by many surgeons, all based on an accurate understanding of facial anatomy and the anatomic changes that occur with aging. More extensive subSMAS dissection has now become a routine part of modern face-lift techniques.

One of Tom’s defining attributes is that he was never satisfied with the status quo and was always ready to learn the newest innovations that would improve results. He readily sought out and adopted the newest laser technology to his resurfacing procedures, traveled to Los Angeles to watch Dr Obaji perform trichloroacetic acid (TCA) peels, and attended symposiums to learn endoscopic surgery. He was quick to recognize innovators and educators and would immediately invite them to Miami to share their experience at Baker Gordon. Many of the aesthetic experts made (or broke) their reputation in Miami based on the requirement of not only having to lecture and show their best results but also demonstrate live how to execute a procedure while under the examination of their peers.

During his career, Tom remained at the academic forefront of aesthetic surgery and fought for university-based surgeons to recognize the relevancy and importance of aesthetic procedures to our specialty. His academic accomplishments, which included writing over 100 articles and several textbooks, allowed him to become academically recognized and accepted, and he was one of the few aesthetic surgeons of his era to serve as a director (and later vice chairman) of the American Board of Plastic Surgery as well as an associate editor of PRS. He also was a founding member of the American Society for Aesthetic Plastic Surgery (known until recently as ASAPS) and served as ASAPS President in 1981–1982, which, he felt, was one of his greatest accomplishments. His ASAPS president’s gavel remained one of the centerpieces of his office, in full display for all visitors and patients.

Tom and I were in practice together for 15 years and for me those years have always felt like my aesthetic surgery fellowship. He encouraged me to write journal articles, encouraged me to lecture and learn from other experts in our field, and mentored me in terms of the organizational duties of Society leadership. Even after we separated, Tom encouraged me to continue to remain an educator and his greatest demand was that I would one day be the President of The Aesthetic Society, the organization that he helped found. He encouraged me to continue as Chair of the Baker Gordon Symposium, and, in respect to Tom’s legacy, I have done my best to continue the tradition of excellence he brought to Miami in 1967.

Tom’s mission in plastic surgery was to bring academic credibility to aesthetic surgery. I think he was fortunate in that he realized the fruition of his efforts during his lifetime and was gratified to see the global embrace of aesthetic surgery as a credible and important subspecialty of plastic surgery. Even a decade into retirement, he attended every ASAPS meeting, staying in the meeting hall until adjournment, remaining interested, and remaining involved.

Tom lived a full life, traveled the world, skied the Alps in Switzerland and Italy, went scuba diving throughout the Caribbean, flew his airplane frequently to the Bahamas, and played poker weekly. Despite all his varied interests, his true love remained plastic surgery. A humble and quiet man, he was well loved and widely respected.

I was greatly blessed to have such a mentor and friend as Thomas J. Baker. He made an incredible difference to my life and surely if I had not known him, my path in plastic surgery would have been much different. With gratitude, I recognize how fortunate I was to know this wonderful man. While it is with great sadness that I write this tribute, I celebrate the many wonderful experiences I shared with Tom and rejoice in a life well lived.

## References

[1] Baker TJ. Chemical face peeling and rhytidectomy. A combined approach for facial rejuvenation. Plast Reconstr Surg. 1962;29:199. 10.1097/00006534-196202000-00007 13864170

[2] Baker TJ, Byrd HS, Barton Jr FE, Hester TR. Approaches to midface and cheek lifting. Aesthet Surg J. 1996;16(1):4-8.

[3] Stuzin JM, Baker TK, Gordon HL, Baker TM. Extended SMAS dissection as an approach to midface rejuvenation. Clin Plast Surg. 1995;22(2):295-311. 7634739

